# Body Mass Index and Self-Perception of Overweight and Obesity in Rural, Urban and Rural-to-Urban Migrants: PERU MIGRANT Study

**DOI:** 10.1371/journal.pone.0050252

**Published:** 2012-11-28

**Authors:** Christian Loret de Mola, Timesh D. Pillay, Francisco Diez-Canseco, Robert H. Gilman, Liam Smeeth, J. Jaime Miranda

**Affiliations:** 1 CRONICAS Center of Excellence in Chronic Diseases, Universidad Peruana Cayetano Heredia, Lima, Peru; 2 Programa de Pós Graduação em Epidemiologia, Centro de Pesquisas Epidemiologicas, Universidade Federal de Pelotas, Pelotas, Brazil; 3 Grupo Latinoamericano de Investigaciones Epidemiológicas, Organización Latinoamericana para el Fomento de la Investigación en Salud, Bucaramanga, Colombia; 4 Medical School, University College London, London, United Kingdom; 5 Department of International Health, Johns Hopkins Bloomberg School of Public Health, Baltimore, Maryland, United States of America; 6 Área de Investigación y Desarrollo, A.B. PRISMA, Lima, Peru; 7 Faculty of Epidemiology and Population Health, London School of Hygiene and Tropical Medicine, London, United Kingdom; 8 Department of Medicine, School of Medicine, Universidad Peruana Cayetano Heredia, Lima, Peru; University of Utah, United States of America

## Abstract

**Objective:**

This study aimed to compare self-reported weight and body mass index (BMI) in order to determine discrepancies between subjective and objective obesity-related markers, and possible explanatory factors of overweight and obesity underestimation, in urban, rural and migrant populations.

**Materials and Methods:**

Data from the PERU MIGRANT study, a cross-sectional study, in low-income settings, of urban, migrant (rural-to-urban), and rural groups, including BMI, self-reported weight and socio-demographic indicators were analyzed. Percentage of concurrences between BMI and self-reported weight and Kappa coefficients for inter-rater agreement were calculated. Univariate and standardized descriptive analyses were performed to identify potential explanatory variables for weight underestimation in only overweight and obese individuals, using established BMI and waist circumference cut offs.

**Results:**

983 Participants–199 urban, 583 migrants and 201 rural–were analyzed. Based on BMI, overall prevalence of obesity was 20.1% (95% CI 17.6%–22.6%), and overweight was 38.3% (95% CI 35.2%–41.2%), with differences between study groups (p<0.001). Only 43% of the whole sample had matching self-reported weight and BMI status, whereas 54% underestimated and 3% overestimated their BMI category. Kappa coefficient, between BMI and self-reported weight, for the entire sample was 0.16, rural residents had the lowest coefficient (0.01) and the most underestimation, especially in the overweight category. In overweight and obese individuals, deprivation index (p = 0.016), age (p = 0.014) and waist circumference (p<0.001) were associated with weight underestimation.

**Discussion:**

Overall, high levels of overweight, obesity, and underestimation of BMI status were found, with poor agreement between BMI and self-reported weight, showing the unawareness of weight status severity in this low-income setting.

## Introduction

The global prevalence of obesity has reached epidemic proportions [1], with rates increasing fastest in developing countries [2] as a result of urbanization, changes in diet and reductions in physical activity [3], [4]. What data is available in Peru shows that prevalence of overweight and obesity in women combined, has increased from 45.7% to 51.5% in urban groups and 33.6% to 36.3% in rural groups between 1991–2 and 2005 [5]. Data on BMI and waist circumference also shows considerably higher figures in urban than rural areas [4]. Prevalence of related non-communicable disease is on the increase in Peru, with deaths from cardiovascular disease and diabetes increased between 2000 and 2007 from 92.9 to 97.1 and 11.6 to 12.8 per 100,000 respectively [6].

Misperception of weight is likely a substantial hurdle to overcome when attempting to target weight-loss [7]. Its better understanding may aid future attempts to tackle the obesity epidemic with interventions, particularly in transitioning societies. While evidence shows that Hispanics in the US are less likely to see themselves as overweight than Caucasians or non-Hispanic white subjects [7], [8], the literature on self-perception of weight in Latin America is scant. A study in Brazil found that perceptions of weight category were not aligned with nutritional status and that underestimation of weight was more common in men, and overestimation more common in women [9]. A study of social class and obesity in six Peruvian cities supported the observation of a gender-bias in weight estimation, and shows a significant gradient by socio-economic status, with individuals in the low and middle socioeconomic categories more likely to underestimate than those in the higher groups [10]. This study, however, was focused in urban areas and, as shown before, rural areas in the country are also facing increases in the obesity epidemic that merits attention [5], and as it has been shown by Darlhy et al, that place of residence, specifically living in a less urban environment, modifies the effect of Socioeconomic status (SES) on overweight [11].

The PERU MIGRANT (Peru's Rural to Urban MIGRANTs) study [12] –for which prevalence of overweight and obesity have been reported elsewhere [4]– provides the opportunity to explore self-perceptions of obesity in a sample of rural, urban and rural-to-urban migrant populations. This study aimed to compare self-reported weight and BMI in order to determine levels of discrepancies between subjective and objective obesity-related markers in the whole sample, as well as in relation to rural, urban or rural-urban migrant status, and potential explanatory variables for underestimation in a subgroup of overweight and obese individuals.

## Materials and Methods

### Setting

In Peru, a high level of rural-to-urban migration took place during the 1970s, 1980s and 1990s, due to an intense period of Political violence, that led to more than 69,000 deaths in rural areas and approximately 120,000 displaced families [13]. The Andean department of Ayacucho was the most affected region, where more than half of the deaths occurred, and of the 2.9 million migrants in the province of Lima in 2007, 6.8% were from Ayacucho, the fourth highest represented district [14].

A shantytown in Lima, “Las Pampas de San Juan de Miraflores”, was selected as the urban site and the village of San Jose de Secce, located in the Huanta province, Ayacucho was selected as the rural site. A detailed protocol for the PERU MIGRANT study, describing rationale and design, has been published in an open access source elsewhere [12].

### Study Design and Participants

The PERU MIGRANT study was a cross sectional study conducted in 2007, with the aim to establish the effect of migration on cardiovascular risk factors [12]. The original study sample consisted of 989 individuals: 199 urban, 589 rural-urban migrant and 291 rural participants. The rural group included permanent residents of the village of San Jose de Secce born in Ayacucho who were not migrants who returned to their village. Migrants were those born in Ayacucho who moved to the city of Lima and were permanent residents of the urban site, and the urban dwellers were individuals who were born in Lima and were permanent residents of the urban site.

A single-stage random sampling based on a census conducted in the urban and rural settings was used. Permanent residents with an age of 30 years or over were considered eligible. Pregnant women and anyone unable to understand and give written consent were excluded. Overall participation rate in the PERU MIGRANT study at enrollment was 73.2%. Detailed information on the participation rates and flowcharts per group have been previously published and are available online [12].

### Study Variables

Each participant answered a detailed questionnaire regarding medical history, lifestyle and socioeconomic indicators. Weight and height, were measured using standardized methods. The question for self-reported weight, asked before the participants were weighed, was: “*For your age, do you consider your weight to be…?*” (originally in Spanish: “*¿Para su edad, usted considera que su peso es…?*”). Answers were limited to four categories: low weight (*bajo peso*), normal (*normal*), overweight (*sobrepeso*), or obese (*obeso*). Correspondingly, BMI, defined as weight divided by quadratic height (Kg/mts^2^), was also grouped into low weight (<18.5 Kg/m^2^), normal (≥18.5 to <25 Kg/m^2^), overweight (≥25 to <30 Kg/m^2^) and obese (≥30 Kg/m^2^), as defined by the World Health Organization (WHO). Other variables used were, sex, age (in 5 year categories from 30–34 to ≥60 years); place of residence (rural, urban, migrant); waist circumference (normal or high defined as greater than and less than 102 cm in males and 88 cm in females respectively as defined by the WHO [15]); self-reported health (poor or very poor, fair, good or very good); and socioeconomic status variables: total family income per month (<US$150, US$150–250, >US$250 dollars), possessions weighted asset index (in tertiles), educational attainment (none/incomplete primary, complete primary, some secondary or higher), parents’ educational attainment (none, incomplete primary, complete primary or higher). The deprivation index is an aggregated measure of household income, asset possession, educational attainment and overcrowding (three or more people per room in house). This deprivation index is binary, with yes referring to a participant in the bottom category of two or more of the four deprivation variables, and no to all others.

### Statistical Analysis

A descriptive analysis included a calculation of the percentage agreement between BMI and self-reported weight (SRW), stratified and total prevalence of Obesity, Overweight, Normal weight, and Low weight, and SRWs for each category of BMI, as well as Kappa coefficients for inter-rater agreement between subjective and objective obesity-related markers. BMI medians and their inter quartile ranges (IQRs) were estimated for all overweight and obese individuals who accurately estimated or underestimated their BMI category. Medians were preferred due to non-normal distribution of BMI in the studied categories.

A sub-analysis of underestimation in overweight and obese individuals by BMI was performed to identify possible explanatory variables, since underestimation in overweight and obese participants by BMI cut-offs is of most importance to public health. Weight understimators were those overweight and obese individuals who self-reported their weight as normal or as overweight, respectively (one category underestimation). In these weight underestimators, the explanatory variables explored were sex, age, place of residence (urban, migrant, rural), deprivation index, self-reported health status, and waist circumference. The socioeconomic status variables: asset possession, education, family income and parental education, were only used for description of the populations. Deprivation index was used to explore association with underestimation as it was seen as a good summary of current socioeconomic status.

Since high waist circumference is also of main importance for obesity-related conditions [15], a separate sub-analysis of potential explanatory variables for underestimation in people with high waist circumference was also explored. In this case, underestimation was defined as those who self-reported their weight as normal or underweight. A stratified descriptive analysis was performed to assess the distribution of underestimation prevalence between each group in each of the other socio-demographic variables.

Fisher exact test was used to explore the association between variables and total underestimation of weight, also trend analysis were performed, a p<0.05 was considered significant, and 95% Confidence Intervals (CI) were reported when needed. Neither a more complex multivariate analysis nor a Mantel-Hansen approximation to explore confounding or interactions between groups were used because of the small sample in each subgroup. Data analysis was made using STATA version 11.2.

### Ethics

Ethical approval for this protocol was obtained from Institutional Review Boards at Universidad Peruana Cayetano Heredia in Peru and the London School of Hygiene and Tropical Medicine in the UK. All enrolled participants gave written informed consent.

## Results

### Description of Participants

A total of 983 participants (53% women), with mean age 48 years-old, were analyzed. Of these, 199 were urban, 583 were migrants and 201 were rural-dwellers. Approximately half of the total participants (45%) had a family income between US$150–$250 dollars per month and 51% had at least some secondary education. There were substantial differences between places of residence in some of these variables. In the rural group, 89% earned less than US$150 dollars per month, and 98% were in the lowest tertils of assets. The urban and migrants were in the middle or highest tertiles of income and assets. Sixty-six percent of rural dwellers had none or incomplete primary education, while 82% of urban and 52% of migrant dwellers had at least partial secondary education. A similar profile was observed with parents’ education, where 81% of the urban group had at least one parent with complete primary education or better, and 53% of the rural group had parents with no education combined. Overall, 60% of participants reported their health as fair, a similar finding across all groups. However, in the rural group 32% thought their health to be poor or very poor, whereas 39% of urban and 28% of migrant dwellers thought their health good or very good.

Overall prevalence of obesity was 20.1% (95% CI 17.6%–22.6%), with a gradient observed by study group: 34.2% (95% CI 27.6%–40.8%) in urban, 21.3% (95% CI 17.8%–24.4%) in migrant and 3% (95% CI 0.6%–5.3%) in rural group (p<0.001). Overall prevalence of overweight was 38.3% (95% CI 35.2%–41.2%), with differences between study groups (p<0.001): 36.7% (95% CI 30%–43.4%) in urban, 46.3% (95% CI 42.1%–50.2%) in migrant and 16.4% (95% CI 11.3%–21.6%) in rural group.

### Inter Rater Agreement between BMI and Self-reported Weight

The percentage of agreement between BMI and self-reported weight was 43% in the whole group, while 3% and 54% overestimated and underestimated their weight respectively (Table 1). Urban, migrant and rural groups had similar percentage concurrence: 43.2% (95% CI 36.3%–50.1%), 43.6% (95% CI 39.6%–47.6%), and 40% (95% CI 33%–46.6%) respectively; and underestimation: 52.8% (95% CI 45.8%–59.7%), 52% (95% CI 47.9%–56%), and 59.7% (95% CI 52.9%–66.5%) respectively. Kappa coefficients showed a poor agreement between BMI and self-reported weight for the entire sample 0.16 (95% CI 0.14–0.18). Kappa coefficients for the urban, migrant and rural groups were 0.18 (95% CI 0.18–0.22), 0.15 (95% CI 0.14–0.16), and 0.01 (95% CI 0–0.06) respectively.

**Table 1:** Percentages of Agreement, Underestimation, and Overestimation between BMI and Self-Reported Weight (SRW).

		BMI n (%)				
		Low weight	Normal	Overweight	Obese	Total
SRW	Low Weight	5 (71.4)	137 (34.1)	31 (8.2)	3 (1.5)	176 (17.9)
	Normal	2 (28.6)	238 (59.2)	166 (44.1)	28 (14.1)	434 (44.2)
	Overweight	0 (0)	27 (6.7)	177 (47.1)	164 (82.8)	368 (37.4)
	Obesity	0 (0)	0 (0)	2 (0.5)	3 (1.5)	5 (0.5)
	Total	7 (100)	402 (100)	376 (100)	198 (100)	983 (100)
	Total Agreement = 43.0%		Total Underestimation = 53.8%		Total Overestimation = 3.2%	

### Discrepancies between BMI Category and Self-reported Weight in the Whole Sample

The largest discrepancies between BMI and self-reported weight were found in the urban obese and rural low-weight participants (Table 2). While 34% of the urban group was classified as obese by BMI, none self-reported as obese and while only 1% of the rural group was underweight by BMI, 49% self-reported as underweight. Discrepancies between the ‘normal’ and ‘overweight’ categories of BMI and self-reported weight were larger in the rural group than the migrant or urban groups. A detailed description of the sample’s BMI and self-reported weight is given in Table 1 and Table 2.

**Table 2:** Number (%) of People by Weight Category according to BMI and Self-Reported Weight (SRW) by Place of Residence.

	Urban n (%)		Migrant n (%)		Rural n (%)	
	BMI	SRW	BMI	SRW	BMI	SRW
Low Weight	2 (1.0)	14 (7.0)	3 (0.5)	64 (10.9)	2 (1.0)	98 (48.8)
Normal	56 (28.1)	76 (38.2)	186 (31.9)	263 (45.1)	160 (79.6)	95 (47.3)
Overweight	73 (36.7)	109 (54.8)	270 (46.3)	251 (43.1)	33 (16.4)	8 (3.9)
Obese	68 (34.2)	–	124 (21.3)	5 (0.9)	6 (3.0)	–

**Figure 1:**
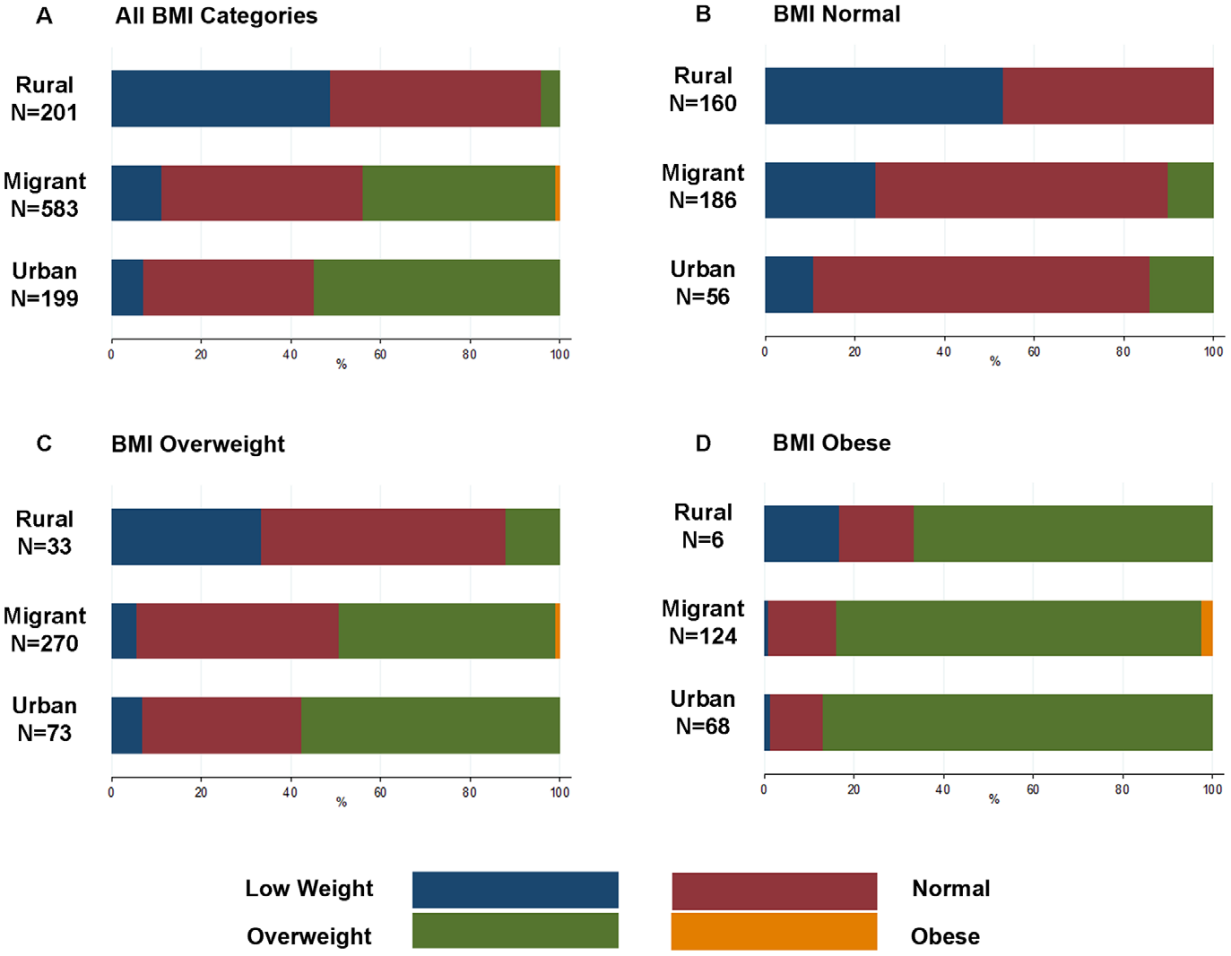
Self-Reported Weight Classification by BMI Categories in Urban, Migrant, and Rural Individuals. A) Self-reported weight of participants in all BMI categories n = 7. B) Self-reported weight of people with normal BMI, n = 402. C) Self-reported weight of people with overweight by BMI, n = 376. D) Self-reported weight of people with obesity by BMI, n = 198.

Figure 1 shows the percentages of participants that self-reported as under-weight, normal, overweight and obese in each BMI category. In total, none of the rural and urban groups’ self-reported obesity and only 5 migrants did, from which 2 were overweight and 3 were obese by BMI. Fifteen percent of the obese group reported their weight as low or normal. In the overweight category, 88% of rural, 51% of migrant and 43% of urban dwellers perceived themselves as having normal or low weight.

### Underestimation in Overweight and Obese Individuals by BMI

In this sub-analysis, the proportion of underestimation was 84.6% (95% CI 70.4%–98.8%) in the rural group 66.9% (95% CI 58.7%–75.2%]) in the urban and 62.5% (95% CI 57.4%–67.5%) in the migrant (p = 0.06). In general, underestimation seemed higher among rural dwellers but similar between urban and migrants (Table 3).

**Table 3:** Weight Underestimation in Overweight and Obese Individuals by Place of Residence and Socio-Demographic Variables^a^.

			*Underestimation n (%)*				
Socio-Demographic variables		n	Urban n = 127	Migrant n = 357	Rural n = 26	Total n = 510	p^b^
Sex	Male	217	37 (67.3)	92 (61.3)	11 (91.7)	140 (64.5)	0.999
	Female	293	48 (66.7)	131 (63.3)	11 (78.6)	190 (64.9)	
Age	30–34	65	4 (25.0)	21 (50.0)	6 (85.7)	31 (47.7)	0.014
	35–39	79	12 (57.1)	32 (62.8)	6 (85.7)	50 (63.3)	
	40–44	82	12 (70.6)	34 (56.7)	4 (80.0)	50 (61.0)	
	45–49	77	13 (72.2)	40 (71.4)	2 (66.7)	55 (71.4)	
	50–54	81	10 (52.6)	38 (63.3)	2 (100)	50 (61.7)	
	55–59	66	20 (90.9)	30 (68.2)	0	50 (75.8)	
	60>	60	14 (100)	28 (63.6)	2 (100)	44 (73.3)	
Deprivation Index	No	429	81 (66.9)	182 (60.5)	5 (71.4)	268 (62.5)	0.016
	Yes	81	4 (66.7)	41 (73.2)	17 (89.5)	62 (76.5)	
Self-Reported Health	Fair	323	52 (67.5)	146 (62.9)	13 (92.9)	211 (65.3)	0.909
	Poor, very poor	49	5 (62.5)	23 (63.3)	3 (60.0)	31 (63.3)	
	Good, very good	137	28 (66.7)	53 (60.2)	6 (85.7)	87 (63.5)	
Waist Circumference	Normal	296	27 (45.0)	119 (55.1)	18 (90)	164 (55.4)	<0.001
	High	165	57 (86.4)	104 (73.2)	4 (66.7)	165 (77.5)	

a Prevalence of Underestimation in one category of weight in Overweight and Obese Individuals by BMI, Stratified by Place of Residence and Socio-demographic Variables, and Univariate Analysis of Total Underestimation for each Socio-demographic Variable.

b Fisher exact test for total underestimation for each socio-demographic variable.

There was no evidence of differences in underestimation in BMI status by sex and self-reported health categories. Age (p = 0.014), deprivation index (p = 0.016) and waist circumference (p<0.001) were associated with underestimation (Table 3). Prevalence of underestimation in general was bigger in urban and migrant dwellers with high rather than normal waist circumference and the opposite was seen in the rural group (Table 3).

### BMI Medians by BMI Group and Self-reported Weight

Using BMI to define weight category, BMI medians were 32.1 Kg/m2 among all obese individuals (IQR 30.9–34.8; n = 198); 32.2 in obese individuals who self-reported as overweight (IQR 31.0–34.9; n = 164); 41.4 in obese subjects who accurately self-reported their weight as obese (40.2–42.6; n = 3); 27.2 in all overweight individuals was (26.1–28.2; n = 377); 26.7 in overweight individuals who self-reported as normal (25.8–27.9; n = 166); and 27.5 in individuals who accurately self-reported their weight as overweight (26.6–28.5; n = 177).

### Underestimation in Overweight and Obese Individuals by Waist Circumference

A total of 242 participants had a high waist circumference (24.6%), from this 211 were women (87.2%), and a gradient of high waist circumference was seen over the place of residence (p<0.001), having a prevalence of 3.5% in the rural, 27.3% in migrants, and 37.9% in urban populations.

The proportion of self-reported underweight or normal weight in the group with high waist circumference was 28.6% (95% CI 0%–64.9%) in the rural group; 18.7% (95% CI 9.7%–27.6%) in the urban group; and 23.1% (95% CI 16.5%–29.7%) in the migrant group, with no significant difference (p = 0.610). There was no evidence of differences in underestimation by sex, nor by self-reported health categories, or deprivation index. Only age (p = 0.009) was associated with underestimation in the whole sample (Table 4).

**Table 4:** Weight Underestimation in Individuals with High Waist Circumference by Place of Residence and Socio-Demographic Variables^a^.

			*Underestimation n (%)*				
Socio-Demographic variables		n	Urban n = 7	Migrant n = 160	Rural n = 75	Total n = 242	p^b^
Sex	Male	31	2 (15.4)	4 (23.5)	1 (100)	7 (22.6)	0.999
	Female	211	12 (19.4)	33 (23.1)	1 (16.7)	46 (21.8)	
Age	30–34	18	1 (16.7)	1 (10.0)	0	2 (11.1)	0.009
	35–39	31	0	5 (23.8)	1 (100)	6 (19.4)	
	40–44	40	1 (9.1)	2 (7.4)	0	3 (7.5)	
	45–49	41	2 (14.3)	3 (11.1)	0	5 (12.2)	
	50–54	38	3 (30.0)	9 (33.3)	0	12 (31.6)	
	55–59	43	5 (33.3)	9 (32.1)	0	14 (32.6)	
	60>	31	2 (20.0)	8 (40.0)	1 (100)	11 (35.5)	
Deprivation Index	No	195	13 (18.8)	27 (22.0)	1 (33.3)	41 (21.0)	0.556
	Yes	47	1 (16.7)	10 (27.0)	1 (25.0)	12 (25.5)	
Self-Reported Health	Fair	155	8 (15.7)	25 (25.0)	1 (25.0)	34 (21.9)	0.141
	Poor, very poor	35	2 (25.0)	2 (8.0)	0	4 (11.4)	
	Good, very good	51	4 (25.0)	10 (29.4)	1 (100)	15 (29.4)	

a Prevalence of Underestimation of Weight in individuals with High Waist Circumference, Stratified by Place of Residence and Socio-demographic Variables, and Univariate Analysis of Total Underestimation for each Socio-demographic Variable.

b Fisher exact test for total underestimation for each socio-demographic variable.

As observed in Table 3 and Table 4, there are differences in the absolute numbers of individuals who qualified as overweight and obese by BMI and high waist circumference. According to BMI (Table 3) there were a total of 510 people in the sample with overweight and obesity (51.9% of total sample), but only 242 (24.6% of total sample) with high waist circumference (Table 4). The absolute number of subjects classified as overweighed or obese by BMI, compared to waist circumference classification, dropped from 127 to 7 in urban and from 357 to 160 in migrants group, but in the rural group it increased from 26 to 75.

## Discussion

### Main Findings

In comparing self-perceived weight with BMI categories, this study found that 43% of participants perceived their weight correctly, 54% underestimated their weight and 3% overestimated. Of the total of 198 obese individuals in the study, only 5 perceived themselves as obese. Of the total of 376 overweight individuals, 196 reported themselves as normal or underweight, with 90% of the rural overweight group underestimating their weight. Not surprisingly, a poor agreement between self-reported weight and BMI was observed. Amongst overweight and obese people by BMI, older, more deprived participants, and those with high waist circumference appeared to underestimate their weight more than their counterparts. Among participants with high waist circumference, only age was related with underestimation, with older participants underestimating more than younger participants.

BMI and waist circumference cut-offs applied to our sample yielded different number of people classified with normal or above normal, a finding supported in other studies [16], [17].

### Comparison with Literature

The levels of underestimation and overestimation of weight found in this study, 54% and 3% in the whole sample respectively, are similar to that shown in a recent study in cities in Peru [10] where 51% of the sample underestimated their weight, and only 4% overestimated it. That study found higher underestimation in men, not replicated in our study, though also an increased risk of underestimation in lower socioeconomic status group, similar to that found here for socioeconomic deprivation. Our figures are higher for underestimation and lower for overestimation than findings from other studies in Brazil –22.6% and 12.2% respectively in one study of adolescents [9] – and outside Latin America [7], [8], [18], [19]. But even though the numbers are not similar, taken together, findings support the idea that there is a population-level discrepancy between BMI and self-perception of weight, oriented towards weight underestimation.

Our results showed that age is positively associated with risk of underestimation of overweight and obesity in the whole sample, a finding supported in other studies in different settings [18], [19]. We also found that Deprivation and SES was associated with underestimation in overweight and obese participants by BMI. Care should be taken with this relationship since it has been shown that the association between SES and obesity changes depending on the SES/obesity indicator used (Education, Assets, Income, etc.) [11], [20]. Furthermore, place of residence modifies the way in which SES affects overweight; in rural areas the relationship might be direct and in urban areas it may be inversed [11]. Further studies should aim to determine which specific SES indicators affects underestimation and how place of residence might modify the effect.

### Theories Explaining Underestimation

It is widely recognized that perceptions of body image are determined –at least in part– by culture [21], [22]. Most studies regarding cultural influences on body image have tended to compare ethnic groups in the US [23], [24]. The terms overweight and obese have complex significance outside the medical world, incorporating social and psychological dimensions and being ‘emotionally balanced’ [25]. A high prevalence of overweight and obesity, 38.3% and 20.6% respectively in our sample, may also have influenced perceptions of weight categories, especially what is ‘normal’. Another factor influencing weight-perception may be the magnitude by which individuals fall into certain BMI cut-offs. In subjects just above the established cut-offs for certain categories, some degree of underestimation may be expected. For example, as evidenced by median BMI, obese people who accurately self-reported as obese had a median BMI of 41.4, while most obese under-estimators were only about 2 Kg/m^2^ units away from the established cut-off of 30 Kg/m^2^ or higher for obesity.

In Peru, national or regional cultural factors may act as drivers of underestimation, though literature on cultural perceptions of food and weight in Peru is weak. A number of studies in other Latin American populations may shed light on these findings [26], [27], [28]. An academic focus on mothers’ perceptions of their children’s nutrition in Mexican populations suggests that a culture exists that views food consumption as an inherently positive thing and that the weight of a child is seen as a surrogate of health [26]. One study of women’s attitudes to health and food in community kitchens in Lima, Peru, described the notion of ‘alimentarse bien’ – to nourish oneself well – as well as a ‘nicely filled out’ body shape as part of a perceived image of health [27]. These phenomena are likely to shape perceptions of food and weight in adulthood, and may be more prominent in poorer groups, potentially explaining the trend observed between deprivation and underestimation. A study comparing Spanish and Cuban women being treated for obesity found that Spanish participants had a more negative outlook on life than their Cuban counterparts, who were optimistic, sociable and virtuous [28].

The above cultural explanations may go some way to explain the trend seen between rural, migrant and urban overweight and obese groups (Figure 1) –and significant increased risk of underestimation in the rural group- with acculturation associated with urbanization acting as a driver of more accurate self-reported weight. However, this may also be explained by higher level of income and education seen in the urban and migrant groups compared to the rural group, two factors that have been shown to promote accurate health estimation in other settings [29], [30].

Equally, self-perceptions of obesity may be affected by the culturally specific meaning of words “overweight” (*sobrepeso*) and “obese” (*obeso*). Peruvian populations could see “overweight” and “obese” as being synonymous terms or inversely could see “obese” as a rare condition, both potentially explaining why the underestimation in obese individuals is so much higher (82.8%) than underestimation in overweight ones (44.1%). The Brazilian population studied by Araujo et al. overestimated more and underestimated less than the Peruvians in this study [9], possibly because the methodology used pictures to classify body image rather than word classification. However, a more educated population was studied in Brazil, which could also explain the difference.

High waist circumference was a predictor of underestimation by BMI group in urban and migrant groups. This finding is counter-intuitive, as one would expect the visual trait of waist circumference to prompt accurate weight estimation, but these finding may be due to the confounding effect of other variables, potentially socioeconomic status, and so should be explored in further studies.

### Strengths and Limitations

The small numbers in some subgroups made sub-analysis difficult, and no further analysis, like stratification by sex – which has been found to be important in weight perception [7], [22] – or multivariate analysis were possible. Significant associations found in our univariate analysis might not have been present in an adjusted multivariable model, because of the possible confounding effect of other variables. Specifically, the high underestimation by BMI status observed in participants with high waist circumference might have been confounded by socioeconomic status or other unmeasured variables.

One should also bear in mind that total prevalence of each category of BMI reported in this study is not representative of all the Peruvian population, since this is a sample from specific locations in Peru with high levels of both rural and urban poverty. Yet the focus on rural-to-urban migration as a distinctive group provides additional insights into the complexities of weight and obesity in a rapidly transitioning setting. Furthermore, other possible predictors could be explored in future research. Finally, the use of BMI as a measure of obesity is subject to some discussion, since it has been shown not to correlate well with visceral fat area [31], correlate less well with metabolic risk factors than waist circumference, and differ by ethnic group [32]. Even though waist circumference could have been a better marker of obesity and overweight, and the risk it represents for developing cardiovascular diseases; the categories of weight used for self-reported could not have been compared in detail, as differentiations between overweight and obese and between normal and low weight are lost.

Furthermore, as discussed earlier, the results of this study could be affected by the cultural specific definition of “obese”, which may differ both between countries and between urban, migrant and rural groups in our study. It would be appropriate for newer quantitative and qualitative studies to explore with greater detail such differences.

Despite these limitations, to our knowledge, this study represents the first attempt to describe self-perception of weight and BMI in urban, rural-to-urban migrant and rural populations in Latin America, and presents the first evidence of a possible association between place of residence and underestimation of weight in a Latin American setting. Also the relatively large total sample size and multiple factors studied provides the opportunity to explore a new field and create a strong base for future research.

### Implications for Public Health

This study has implications for current public health interventions aimed at reducing weight in Peru and elsewhere, especially in low socioeconomic settings like the one studied, where we found that levels of overweight and obesity is high, and over two thirds of these individuals, a group to be targeted with such interventions, are unaware of the severity of their weight status. Since accurate self-perception of overweight and obesity is associated with attempts to lose weight [7], [33], [34], this presents a considerable group that is unlikely to seek lifestyle changes, to listen to dietary advice or adopt strategies to reduce their weight.

We emphasize the implications of these results, which are from predominantly low-income groups. Studies have shown that there may be a global shift in obesity coupled with the nutrition transition, especially in the western hemisphere [10], [35], [36]. In countries like Peru, where an economic transition is taking place, we may see the positive relationship between obesity and income be inverted [35], as has been seen in similar settings, mainly for urban populations [11]. In the light of our results, new efforts in these vulnerable populations are needed, where obesity is increasing while awareness is low. Evidence from this cross-sectional study suggests that underestimation of weight is particularly prevalent in Peru, in rural, migrant and urban populations.
